# Biological sex classification with structural MRI data shows increased misclassification in transgender women

**DOI:** 10.1038/s41386-020-0666-3

**Published:** 2020-04-09

**Authors:** Claas Flint, Katharina Förster, Sophie A. Koser, Carsten Konrad, Pienie Zwitserlood, Klaus Berger, Marco Hermesdorf, Tilo Kircher, Igor Nenadic, Axel Krug, Bernhard T. Baune, Katharina Dohm, Ronny Redlich, Nils Opel, Volker Arolt, Tim Hahn, Xiaoyi Jiang, Udo Dannlowski, Dominik Grotegerd

**Affiliations:** 1 Department of Psychiatry, University of Münster, Albert Schweitzer-Campus 1, A9, 48149 Münster, Germany; 2 Department of Computer Science, University of Münster, Einsteinstraße 62, 48149 Münster, Germany; 3 Department of Psychiatry and Psychotherapy, Agaplesion Diakonieklinikum, 27356 Rotenburg, Germany; 4 Department of Psychology, University of Münster, Fliednerstraße 21, 48149 Münster, Germany; 5 Department of Epidemiology and Social Medicine, University of Münster, Albert Schweitzer-Campus 1, D3, 48149 Münster, Germany; 6 Department of Psychiatry and Psychotherapy, University of Marburg, Marburg, Germany; 7 Department of Psychiatry, Melbourne Medical School, The University of Melbourne, Parkville, VIC Australia; 8 The Florey Institute of Neuroscience and Mental Health, The University of Melbourne, Parkville, VIC Australia

**Keywords:** Predictive markers, Neuroscience

## Abstract

Transgender individuals (TIs) show brain-structural alterations that differ from their biological sex as well as their perceived gender. To substantiate evidence that the brain structure of TIs differs from male and female, we use a combined multivariate and univariate approach. Gray matter segments resulting from voxel-based morphometry preprocessing of *N* = 1753 cisgender (CG) healthy participants were used to train (*N* = 1402) and validate (20% holdout *N* = 351) a support-vector machine classifying the biological sex. As a second validation, we classified *N* = 1104 patients with depression. A third validation was performed using the matched CG sample of the transgender women (TW) application sample. Subsequently, the classifier was applied to *N* = 26 TW. Finally, we compared brain volumes of CG-men, women, and TW-pre/post treatment cross-sex hormone treatment (CHT) in a univariate analysis controlling for sexual orientation, age, and total brain volume. The application of our biological sex classifier to the transgender sample resulted in a significantly lower true positive rate (TPR-male = 56.0%). The TPR did not differ between CG-individuals with (TPR-male = 86.9%) and without depression (TPR-male = 88.5%). The univariate analysis of the transgender application-sample revealed that TW-pre/post treatment show brain-structural differences from CG-women and CG-men in the putamen and insula, as well as the whole-brain analysis. Our results support the hypothesis that brain structure in TW differs from brain structure of their biological sex (male) as well as their perceived gender (female). This finding substantiates evidence that TIs show specific brain-structural alterations leading to a different pattern of brain structure than CG-individuals.

## Introduction

Being transgender describes the stable feeling of belonging to the opposite sex rather than the biological sex assigned at birth, while the term cisgender (CG) describes the feeling of coherence between biological sex and perceived gender.

Although there is an ongoing social and political debate regarding the terms and phrases used to describe gender, little is known about how a divergence between biological sex and perceived gender emerges. A popular view is that sexual brain differentiation and body development diverge in transgender individuals (TIs) [1]. Evidence for this comes from studies in female infants with congenital adrenal hyperplasia, who develop male playing behavior [2, 3]. Due to prenatally circulating testosterone, the brain of such female infants is structurally organized as a male brain, while their body development is female [1–5].

Previous research provides extensive information on how brain structure differs as a function of biological sex. Briefly, localized sex differences show higher gray matter volume in CG-men, while the volume of limbic structures is particularly increased in CG-women [6]. However, sexual differentiation seems less prominent in the brain compared with physical appearance [7–9]. Hence, brains cannot easily be classified into dimorphic gender categories [10].

Few ROI-based approaches have studied how the brain structure of TIs differs from CG-individuals. Compared with CG-men, transgender women (biological sex male, perceived gender female, TW) show structural alterations of areas associated with body perception. Brain structures that repeatedly showed alterations across multiple studies are the putamen [11] and the insula [12]. However, the alterations are highly heterogeneous in their direction and the reported studies only investigated individuals before cross-sex hormone treatment (CHT). Comparisons between TW-pre/post-CHT with CG-individuals again exhibited heterogeneous results [9, 13–18]. CHT in TW combines treatment with antiandrogens and estradiol and is associated with region-specific structural alterations of the brain [19] such as local volume and cortical thickness decreases [15, 20]. However, longitudinal studies are scarce and a recent large study did not find any differences between TW-pre and post-CHT [9, 16].

Next to univariate analyses, multivariate analyses offer new insights into the similarities and differences between CG and TIs [21, 22]. In contrast to univariate analysis, multivariate analysis does not focus on identifying mean differences between individuals rather than recognizing the discriminative patterns within the data applicable on an individual level. This may be utilized to subdivide data into broader categories, but also to identify cases that exhibit unusual patterns and cannot be categorized easily. This approach is particularly interesting for TI, since they perceive a disparity between their gender and their biological sex. Hence, one could assume that they represent cases that exhibit unusual data patterns, e.g., hormone levels, personality traits or brain function, and structure. Recent studies also show a variety of brain-structural differences between TIs and CG-individuals. Thus, a univariate approach might not be suitable to clarify how TIs and CG-individuals differ from each other structurally.

Another methodological motivation for choosing multivariate techniques is that samples of TIs are usually small. Using a multivariate approach trained and validated on larges samples of CG-individuals and applied to TIs allows more valid conclusions about brain-structural differences between TIs and CG-individuals.

Multivariate analyses have already been used to investigate whether TIs can be separated from CG-individuals by their brain volumetric patterns [21, 22]. Both studies show decreased accuracy in biological sex classification in TIs compared with CG-individuals. However, it has been recently criticized that classifiers trained with small sample sizes often lead to high accuracies, but low external validity [23]. Hence, in contrast to previous studies, we trained and validated a biological sex classifier with large samples of CG-participants without any psychiatric comorbidities. We then applied the classifier to a smaller sample of TW. To ensure that observed misclassification is not caused or biased by psychiatric comorbidity, we performed a second validation of the classifier in an additional large validation-sample with patients with Major Depressive Disorder (MDD). A third validation was performed in a matched CG sample of the TW application-sample, whose data were recorded at the same time and in the same scanner.

Thus, an extensively greater generalizability is expected and therefore real-life applicability is enhanced.

Our hypotheses for the multivariate analysis are:

1. The classifier trained on healthy CG-participants shows significantly worse performance when applied to a sample of TW
2. The classifier trained on healthy CG-participants performs equally well in a validation-sample of CG-patients suffering from major depression
  Following our multivariate approach, we investigated local structural brain alterations in the putamen and the insula [9, 11, 12, 24–26]. Since TW differ in brain structure from both CG-men and -women, with TW exhibiting lower volume in the putamen [12] and insula [9] than CG-men, but lower volume than CG-women [9, 27, 28], we hypothesize that
3. CG-women show lower volume in comparison to CG-men [6].
4. TW-pre and post-CHT show increased volume in comparison to CG-women
5. TW-pre and post-CHT show lower volume in comparison to CG-men
  Since we expect CHT to be associated with a further feminization of brain structure and hence reduced volume, we hypothesize that
6. TW-pre-CHT show higher volume in comparison to TW-post-CHT.

## Materials and methods

To obtain and validate a predictor for biological sex based on structural MRI brain scans, we used three different samples, which purposes are briefly described here prior to sample characteristics: a classifier was trained on a large sample of CG-individuals without any psychiatric disorder using a cross-validation procedure. An independent subsample randomly drawn in advance, served as the first validation set, to avoid overfitting (Supplementary Fig. S1). To rule out that depressive symptoms influence the performance of the predictor in our TW-group, we used a second validation-sample with MDD-patients. Next, the classifier was applied to data from TW-individuals, and to a third validation group whose data were acquired at the same time and with the same scanner as the TW-sample.

### Data

#### Cisgender training sample and first validation set

The data from a sample of *N* = 1753 CG-participants without any evidence of previous psychiatric disorders served as the basis for the training. History of psychiatric disorders was ruled out using the structured clinical interview following DSM-IV criteria [29]. The participants were taken from three different cohorts: the Muenster Neuroimaging Cohort (MNC, *N* = 666 [30]), the BiDirect study (BD, *N* = 434 [31]), and the FOR2107 study (*N* = 653 [32, 33]). Exclusion criteria for the MNC were presence or history of major internal or neurological disorder, dependence on or recent abuse of alcohol or drugs, hypertension, and general MRI contraindications. BD and FOR2107 have similar exclusion criteria; details are described in Supplementary Table S1 and elsewhere [32, 34].

#### Second, clinical validation-sample—patients suffering from major depressive disorder (MDD)

To exclude that potential differences in classification true positive rate are due to comorbid depressive symptoms in TW, data from a clinical sample (*N* = 1404) of patients diagnosed with MDD were used as second validation-sample. Four hundred and fifty MDD patients exhibited psychiatric comorbidities such as anxiety disorders or substance abuse. Diagnoses were again verified with the structural clinical interview according to DSM-IV criteria [29]. The MDD sample consisted of *N* = 285 participants from the MNC, *N* = 591 from the BD study, and *N* = 528 from the FOR2107 study (Supplementary Table S1). Additional exclusion criteria were presence of bipolar disorder, schizoaffective disorders and schizophrenia, substance-related disorders, current benzodiazepine treatment (wash out of at least three half-lives before study participation), and recent electroconvulsive therapy. Nearly all patients were under psychopharmacological antidepressant treatment and/or received psychotherapy.

#### Application: transgender application-sample including third validation-sample

To test for a different classification of CG and TW, we used an independent sample of *N* = 29 TW. Three TW had to be excluded from our analysis due to poor image quality and artifacts. Data of TW were collected in conjunction with a set of CG-controls that serve as the third validation-sample of *N* = 19 CG-women and *N* = 15 CG-men (Transgender study (TSS)). TW were recruited during their treatment at the outpatient clinic of the Department of Psychiatry at the University of Münster. Before treatment and study inclusion all participants were carefully tested for chromosomal abnormalities such as Klinefelter syndrome, screened for personality disorders and other psychiatric comorbidities using the structural clinical interview I and II according to DSM-IV criteria (comorbidities are listed in Supplementary Table S5).

Data of TW and CG were recorded under equal conditions (e.g., scanner, timeframe, study protocol, investigator), ruling out possible confounding of the classifier due to scanner variability. The TW were in different treatment states, with 18 already treated with hormones (Supplementary Table S2). Further details can be found in the original study [35].

#### Image acquisition and structural preprocessing

Image acquisition and structural preprocessing followed previously published protocols for the MNC [36, 37], the FOR2107 [33] and the BiDirect Cohort [31]. A detailed description can be found in Supplementary Methods S1.

### Analysis

#### Multivariate analysis

**Fig. 1:**
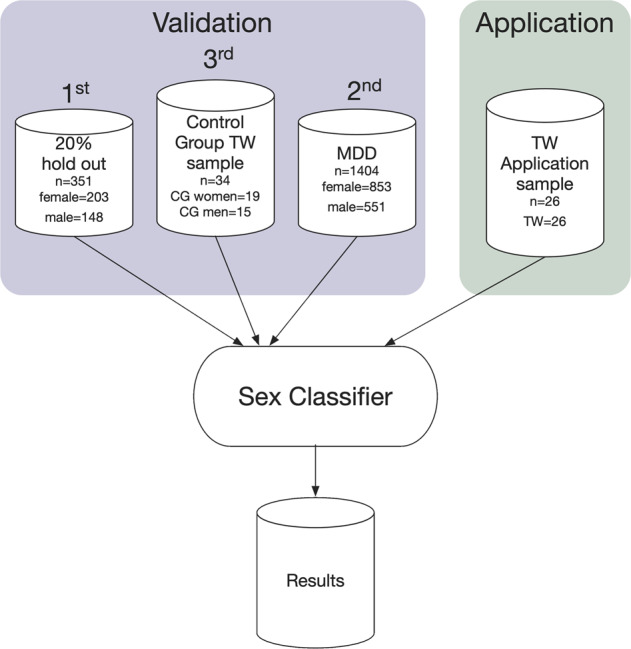
Application of the trained classifier for biological sex prediction. CG cisgender, TW transgender women, MDD major depressive disorder.

Individualized prediction of the biological sex was assessed with a support vector classifier, implemented in the Scikit-learn toolbox [38]. CAT12 whole-brain gray matter images were used as a classifier input [39]. Gray matter images were resliced to a voxel size of 3 × 3 × 3 mm³, to reduce dimensionality while preserving maximal localized morphometric differences. The training process was strictly separated from the evaluation, by selecting a random validation set of 20% (*N* = 351, female = 219, male = 132), which was not used during classifier training and testing. The remaining data set of *N* = 1402 subjects was balanced for sex with a random undersampling procedure (*N* = 1218, female = 609, male = 609), and used in a tenfold split procedure resulting in balanced training sets of 1096 subjects in each fold. A principal-component analysis was performed next, to further reduce the dimensionality of the data. The maximum number of principal components is limited to 1096, the number of subjects resulting from the tenfold split. We carried out a Bayes-statistic-based hyperparameter optimization for the support vector classifier (Scikit-Optimize [40]), nested in the tenfold cross-validation. The parameter search included choice of the kernel (radial basis function (rbf) or linear), the C parameter (10^−2^–10^2^, non-discrete log-scale), which influences penalties for misclassification, and the gamma parameter (10^−6^–10, non-discrete log-sale), influencing the curvature of the decision boundary. In this iterative Bayes approach, a total of 100 parameter combinations were evaluated. Quality and classifier performance are reported by area under the ROC curve (AUC). The classifier resulting from the best combination of hyperparameters was finally determined using our first validation set, the 20% drawn in advance from the original sample. To exclude potential effects of comorbid depression, this step was repeated with the sample of MDD subjects, as a second validation sample (Fig. 1).

The final trained and validated classifier was then applied to the application-sample with TIs. To test if classification results differ between CG-men and TW (same biological sex), we applied the true positive rate (TPR), since balanced accuracy (BACC) is a measure not applicable to one-group-only scenarios. Fisher’s exact test was used to clarify whether TPR differs statistically between samples. Interpretation of TPR is based on the hypothesis that TW belong to the category of male biological sex.

In order to achieve optimal generalization of our classifier, multiple scanners were deliberately incorporated. A specific correction for possible scanner effects was not intended. Instead, the purpose was to establish a classifier based on scanner invariant features given the large amount of training data and expected excellent classification performances. Comparison of the recognition rates between the individual scanners yielded no significant differences. Hence, an influence of the scanner on the classification results could not be detected—supporting our expectation (see Supplementary Table S6). However, it should be pointed out that our data reveal a practically identical classification performance of the classifier trained on the multi-scanner training set (94.01% BACC in the first validation) to its application on the third validation sample (CG-control group of the TW-sample), using a different single scanner environment (94.03% BACC), suggesting that the classifier learned scanner independent features driving the classification performance.

#### Univariate analysis

The methodological details of the univariate analysis can be found in Supplementary Methods S2.

## Results

### Multivariate analysis

#### Cisgender training and first validation sample

The training of the classifier led to two results. The first result was the estimation of a hyperparameter set, determined with the Bayes optimization method. The hyperparameter optimization estimated an rbf kernel, *C* = 27.3 and gamma = 2.4 × 10^−05^ for the SVM as optimal approximation for the present problem.

Based on the estimated hyperparameters, the second result was the classification outcome of the 20% validation set, which provided a performance indication for the trained classifier. The BACC for the validation set classification was 94.01% (Table 1).

**Table 1:** Results of the validation set (*N* = 351; *N*_male_ = 148; *N*_female_ = 203).

Groups	Actual	
	Female	Male
Predicted		
Female	202 (TPR = 99.9%)	17 (TNR = 11.5%)
Male	1 (TNR = 0.1%)	131 (TPR = 88.5%)
Related metrics:		
Accuracy: 94.87%		
Balanced accuracy: 94.01%		
Precision: 99.24%		
Recall: 88.51%		
F1-score: 0.9357		

Classification results in absolute numbers and percentage of accurately identified biological sex.

*TPR* true positive rate (sensitivity), *TNR*  true negative rate (specificity).

Using a Fisher’s exact test the TPR CG-women was significantly increased in comparison to CG-men *p* < 0.001.

The confusion matrix (Supplementary Table S3) revealed that our classifier assigns the female biological sex (TPR = 99.9%) more accurately than the male biological sex (TPR = 88.5%). These results are visualized by a ROC curve, based on the probabilities for a classification as male (Supplementary 2a), with a calculated area under the curve (AUC) of 0.99.

#### MDD second validation sample

To rule out that MDD comorbidity had any influence on the classifier, we used a second validation set consisting of 1404 MDD subjects (853 CG-women, 551 CG-men). Our classifier reached a BACC of 92.06%, and a TPR of 86.93% for CG-men in this sample (Table 2, Supplementary Table S2). The results of the classifier, the corresponding ROC curve (Supplementary Fig. S2d), and the AUC of 0.99 are similar to the results of the first validation set. Fisher’s exact test revealed no significant differences between the distribution of results of the first and second validation-sample (Supplementary Table S6).

**Table 2:** Results of the second validation set (*N* = 1404; *N*_male_ = 551; *N*_female_ = 853).

Groups	Actual	
	Female	Male
Predicted		
Female	829 (TPR = 97.2%)	72 (TNR = 13.1%)
Male	24 (TNR = 2.8%)	479 (TPR = 86.9%)
Related metrics:		
Accuracy: 93.16%		
Balanced accuracy: 92.06%		
Precision: 95.23%		
Recall: 86.93%		
F1-score: 0.9206		

Classification results in absolute numbers and percentage of accurately identified biological sex.

*TPR* true positive rate (sensitivity), *TNR*   true negative rate (specificity).

#### Transgender application sample and cisgender third validation sample

**Fig. 2:**
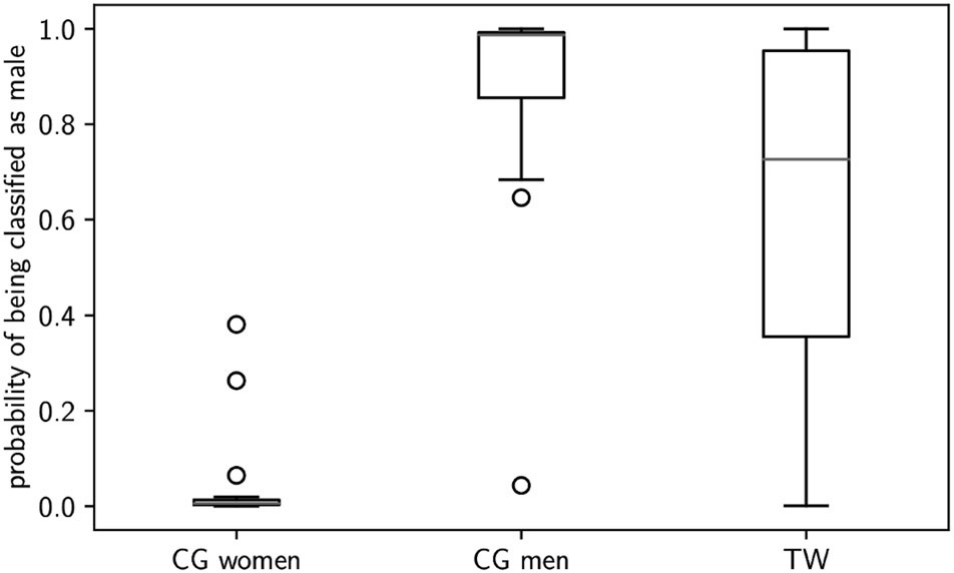
Box plot for the predicted probabilities of male sex based on the application-sample and the third validation-sample, including transgender and cisgender individuals. CG cisgender, TW transgender women.

The BACC for the third validation-sample was 94.03% (CG-part of the TW-sample). The TPR for CG-men was 93.3% and for CG-women 94.7% (Table 3). However, the TPR for the TW was remarkably low at 56% (Supplementary Table S4); see visualization by ROC curves (Supplementary Fig. S2b, c). The corresponding AUC differed as a function of group between 0.99 (CG-men) and 0.95 (TW). This difference in TPR was significant, as Fisher’s exact test showed a statistically significant difference between TPR of CG-men and TW with hormone treatment (Table 4). The output probabilities of the classifier are represented descriptively in Fig. 2, as a box plot.

**Table 3:** Results of the application set (*N* = 60; *N*_cg_men_ = 15; *N*_cg_women_ = 19; *N*_TW_ = 26).

Groups	Actual		
	CG women	CG men	TW
Predicted			
Female	19 (TPR = 100.0%)	1 (TNR = 6.7%)	10 (TNR = 38.5%)
Male	0 (TNR = 0.0%)	14 (TPR = 93.3%)	16 (TPR = 61.5%)
The following metrics are related to the CG groups only:			
Accuracy: 94.12%			
Balanced accuracy: 96.67%			
F1-score: 0.9655			

Classification results in absolute numbers percentage of accurately identified biological sex.

*CG* cisgender, *TW*  transgender women, *TPR*   true positive rate (sensitivity), *TNR*   true negative rate (specificity).

**Table 4:** Classification results in the application sample.

Group	*N*	TPR in % (*N* correct/total)	Fisher’s exact test against CG men
CG women	19	100.00% (19/19)	*p* = 1.0
CG men	15	93.33% (14/15)	–
TW	26	61.54% (16/26)	*p* = 0.028***
TW (treatment naive)	8	87.50% (7/8)	*p* = 0.999
TW (post-CHT)	18	50.00 % (9/18)	*p* = 0.008***

Classification results in percentage of true positive rate identified biological sex.

*TPR* true positive rate (sensitivity), *cg* cisgender, *TW*  transgender women, *CHT * cross-sex-hormone treatment.

***indicates significance of the Fisher’s Z-Test (*p* < 0.05).

### Univariate analysis

**Fig. 3:**
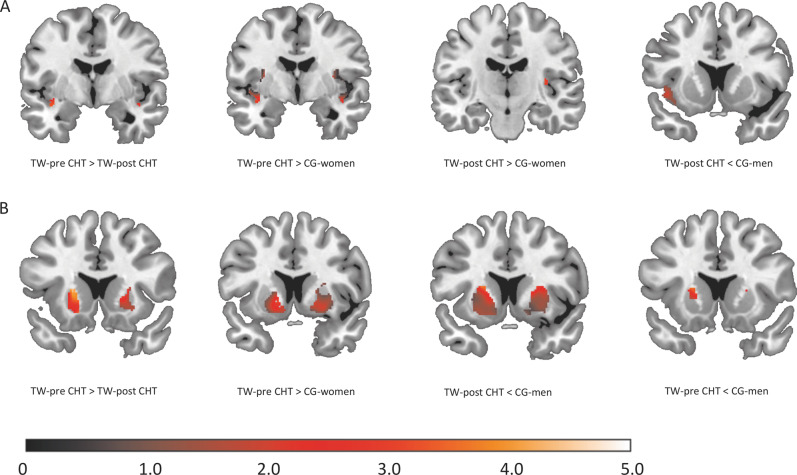
Significant results of the univariate gray matter analysis. Color-bar represents *t*-values of the extracted clusters. Image shows the cluster at the respective peak voxel as reported in Table 3. **a** Alterations of the insula between groups (cisgender men, cisgender women, and transgender women before vs. after hormone treatment). **b** Alterations of the putamen between groups (cisgender men, cisgender women, and transgender women before vs. after hormone treatment) CG cisgender, TW transgender women, pre-CHT before cross-sex-hormone treatment, post-CHT after cross-sex-hormone treatment.

The region of interest analysis is summarized in Table 5 and Fig. 3 (see coordinates and detailed statistics there). Briefly, using rigorous alpha correction, our analysis revealed no differences between TW-post-CHT and CG-women in the bilateral putamen. In the insula, TW-post-CHT showed higher volume than CG-women. TW-post-CHT and CG-women both showed lower volume of the insula and putamen compared with CG-men.

**Table 5:** Results of the univariate gray matter region of interest analysis of the insula and putamen.

Compared groups	Region of interest	Side	TFCE	*p*-FWE	*k*	*x*	*y*	*z*
TW-pre > TW-post	Insula	L	91.50	0.012	76	−38	−3	−12
		R	54.96	0.033	23	32	10	−16
	Putamen	L	466.55	<0.001	2005	−21	16	8
		R	395.31	<0.001	1409	27	−8	15
TW-pre > CG-women	Insula	L	63.21	<0.001	1926	−39	−3	−12
		R	52.58	<0.001	2299	34	15	−10
	Putamen	L	274.31	<0.001	2381	−21	10	12
		R	257.58	<0.001	2316	26	−4	14
TW-pre > CG-men	Putamen	L	203.55	<0.001	892	−21	15	9
		R	183.13	<0.001	576	28	−3	15
TW-post < CG-men	Putamen	L	100.64	0.001	1050	−14	9	−2
		R	70.60	0.001	1429	26	4	−8
	Insula	L	38.69	0.005	303	−42	14	−6
		L	30.99	0.010	124	−42	−8	4
		R	21.37	0.001	131	30	−18	20
TW-post < CG-women	Insula	R	114.58	0.021	99	34	−15	9
CGM > CG-women	Insula	R	109.23	<0.001	1789	39	16	3
		L	49.7	<0.001	1199	−44	14	−8
		L	13.07	0.004	48	−44	−14	8
	Putamen	R	100.11	<0.001	1972	27	6	−4
		L	81.13	<0.001	1509	−26	−4	−3

Table reports respective statistics of significant clusters of the group comparisons between transgender and cisgender individuals. Clusters resulted from group comparisons corrected for total intracranial volume, age, and sexual orientation.

For reasons of brevity no results below a threshold of *k* = 22 voxel have been reported.

*TW* transgender women, *Cg* cisgender, *pre* before hormone treatment, *post* after hormone treatment, *L* left, *R* right, *k* cluster size, *dF* degrees of freedom, *TFCE*  threshold-free-cluster-enhancement with subsequent family-wise-error-correction. Coordinates are reported according to MNI-space.

In contrast, TW-pre-CHT showed larger volume in both ROI analyses compared to CG-women. Interestingly, TW-pre-CHT also showed higher volume in the putamen compared with CG-men.

TW-post-CHT showed lower volume of both regions of interest compared to TW-pre-CHT in both regions of interest. CG-men showed larger volume in both regions of interest compared to CG-women.

Detailed results of our exploratory whole-brain analysis can be found in the Supplementary Table S4. Omitting TW individuals with psychiatric comorbidities did not alter findings in general (see Supplementary Tables S7 and S8). However, conclusions should be made with caution due to limited sample size.

## Discussion

In the present study, we developed an SVM using hyperparameter optimization resulting in an accurate classification of biological sex based on structural MRI images. The classifier, trained on a large training set of healthy CG-individuals, performed equally well in three independent validation samples of healthy CG-individuals, and CG-participants suffering from MDD. When applying the same classifier to structural MRI data of TW, the SVM shows a much lower TPR, resulting in significantly more misclassifications of the biological sex of TW (male) in favor of their perceived gender (female). Moreover, the descriptive statistics of classification probabilities regarding TW (Fig. 2) indicate a pattern of prediction uncertainty that is not observable in CG.

Hence, our results shed light on two important aspects in biological psychiatry of TIs: (1) The impact of hormonal treatment on brain structure, (2) the separation of psychological distress (i.e., depression), hormonal treatment, and trait characteristics of being a TI.

Our results replicate the finding that biological sex is increasingly misclassified in TIs, as previously described [21, 22]. This might encourage further investigations into the cause for increased misclassifications in TW. Most notably and in contrast to previous studies, we could rule out that our findings are biased by comorbid depression and antidepressant medication. Given that the results of the first validation sample of healthy CG-participants were replicated in a large clinical sample of CG-patients suffering from major depression, the classifier is reliable and robust to noise even from psychiatric disorders such as MDD and medication, which have been associated with structural brain changes [41, 42].

Our biological sex classifier shows a higher external validity than other biological sex classifiers. First, it has been tested on controls and MDD-patients, with high and very similar accuracy. Second, the SVM has been trained on large samples that have been collected at different sites. Hence, our SVM can be regarded as more generalizable while preserving performance and accuracy, indicating its robustness to noise.

In the present work, we focused on the first application of this SVM on TW. We observed that our SVM was increasingly inaccurate in TW, compared with healthy CG-controls. The explorative analysis revealed that this inaccuracy was particularly increased in TW who had hormonal treatment.

Although our TW-pre-CHT sample size was low, we aimed to differentiate structural brain alterations between TW-pre and TW-post-CHT as well as in comparison to CG-women and -men. Our results show brain-structural alterations dependent on the treatment state of TW.

Volumes of the insula and putamen were larger in TW-pre-CHT than in CG-women, while TW-post-CHT showed lower volumes of the right insula compared with CG-women.

In comparison to CG-men, TW-pre-CHT showed larger volumes of the putamen, while TW-post-CHT showed lower volumes of both insula and putamen. Thus, TW independent of treatment state show brain-structural alterations in our regions of interest in comparison to both, CG-men and -women.

Detailed analysis of TW-pre compared with -post-CHT revealed a less pronounced pattern of structural brain alterations in TW-post-CHT compared with CG-women. Comparing TW-pre with TW-post-CHT revealed lower volume of TW-post-CHT in both regions of interest, as well as the whole-brain analysis. This implies that CHT induces a further feminization of brain structure in TW. This result fits with previous longitudinal studies that have shown reductions of cortical thickness in TW-pre to post-CHT [26]. Structural and functional alterations of the insula have consistently been associated with TIs compared to CG-individuals [9, 12, 24, 25, 43]. The insula is associated with body and self-perception. Behaviorally, TW perceive an incoherence between their biological sex and perceived gender that is accompanied by altered insula activity in response to bodily sensations [44].

Brain structural alterations of the putamen have been associated with TW across multiple studies and independent of treatment state [11–13]. We examined the putamen volume across different treatment states. Our study reveals that TW-pre show a higher volume of the putamen compared with CG-men and CG-women, while TW-post show lower volume of the putamen compared with CG-men, but not to CG-women. However, it remains unknown how CHT influences these structural alterations of TW. Longitudinal examinations are required to reveal region-specific structural alterations to estimate the impact of CHT of brain structure.

Our combined univariate and multivariate approach revealed associations of CHT with lower accuracy in detecting the biological sex of TW. Our results show that the brain structure of TW aligns with neither their biological sex (male) nor their perceived gender (female). This implies that there is a biological basis for being transgender and thus, destigmatizes TIs. Further, this evidence can be used in psychoeducation during treatment of gender dysphoria. The diagnosis of gender dysphoria is new to DSM-5 to allow for treatment if TIs suffers from distress due to incoherence between perceived gender and biological sex. Our results could relieve distress in transgender patients in case of the experience of guilt or shame due to the discrepancy between biological sex and perceived gender.

In line with this idea, hormonal processes, brain-structural development, and the development of gender identity are intertwined [17]. Intrauterine hormones drive the development of gender identity, rather than social learning processes [45, 46]. The male physical appearance is formed in the first trimester, due to effects of testosterone, and the female body develops due to the lack of androgens in this period [47]. While the maturation of reproductive organs is more or less limited to the first trimester, brain development is continuing throughout pregnancy [4, 48]. Hormonal influences after the first trimester do not change the biological sex, but the experience of gender and thus might be responsible for the incoherence between biological and experienced sex. Since hormonal influences change gender perception as well as brain structure, CHT may lead to misclassifications in the TW-group after treatment. Our univariate data indeed show that CHT is associated with structural brain alterations comparing TW-pre and post-CHT to CG-individuals. A previous study showed increased misclassification of biological sex even in untreated TW [21], which we could not statistically support due to the small sample size of our untreated group (*N* = 8). Therefore, further studies should follow up on this effect, with higher sample sizes of untreated TW to increase power. An extension of the design with a second control group (women with hormonal treatment) should be used to clarify whether misclassification is an effect of treatment only, due to the combination of being transgender and CHT.

The present SVC provides a new tool for research in biological psychiatry. Prevalence of many psychiatric disorders is often higher for one biological sex than for the other. For example, prevalence in autism is higher for biological men than for biological women. Hence, it was hypothesized that female patients with autism might be similar in their brain structure to men. A previous study that developed a biological sex classifier using structural MRI scans and applied it to patients with autism [49] indeed showed increased misclassifications of biological sex in female patients with autism. Therefore, biological sex misclassifications might point to involvement of aberrant biological sex development in the onset of such neurodevelopmental disorders. Future studies could use our trained classifier (https://photon-ai.com/model_repo/bsc_mri) to test for misclassifications in other clinical diagnoses with high gender imbalance in prevalence rates, such as eating disorders, substance use disorders, or anxiety disorders.

### Limitations

Next to our training and validation strategy (visualized in Fig. S1), a variety of other strategies exist such as repeated nested k-fold cross validation (see also [22]). The latter is an adequate means of choice in the absence of external validation samples and produces robust estimates. However, even by preserving similar classification performances, we cannot rule out that other validation strategies could result in learning other patterns and therefore influence the prediction on TW individuals. In addition, due to our small sample size of TW, replication of the prediction failure of our SVM in TIs pre and post-CHT is needed. To verify that our effect is due to hormonal treatment, larger samples and studies in transgender men (biological sex female) are needed. Future studies should further dissect effects of gender dysphoria from depression, and effects of hormonal treatment from the state of being a TI.

Finally, on the basis of the present data, we cannot draw firm conclusions on why the sensitivity of our classifier is greater towards the female. Further research is needed that investigates how classification performance in CG-men and -women is associated with sex hormones.

## Conclusions

In this study, we present a highly accurate biological sex classifier in CG-individuals that shows a significantly decreased accuracy in TIs after CHT. Our results underline that the brain structure of TIs is similar to both, the brain structure of their perceived gender and biological sex. This implies that brain structure of TW differs from both CG-men and -women. Based on our brain-structural data, we suggest a dimensional rather than binary gender construct which will contribute to the destigmatization of TIs.

## Funding and disclosure

This work was funded by the German Research Foundation (DFG, grant FOR2107 DA1151/5–1 and DA1151/5–2 to UD; SFB-TRR58, Projects C09 and Z02 to UD) and the Interdisciplinary Center for Clinical Research (IZKF) of the medical faculty of Münster (grant Dan3/012/17 to UD). The BiDirect Study is supported by a grant of the German Ministry of Research and Education (BMBF) to the University of Muenster (01ER0816 and 01ER1506).

Biomedical financial interests or potential competing interests: TK received unrestricted educational grants from Servier, Janssen, Recordati, Aristo, Otsuka, neuraxpharm.

The other authors (CF, KF, SAK, CK, PZ, KB, MH, IN, AK, BTB, KD, RR, NO, VA, TH, XJ, UD, DG) declare no competing interests.

## Supplementary information

Supplemental Material

Figure S1

Supplementary Figure S2
