# Correction to: Biological sex classification with structural MRI data shows increased misclassification in transgender women

**DOI:** 10.1038/s41386-021-01203-2

**Published:** 2021-10-27

**Authors:** Claas Flint, Katharina Förster, Sophie A. Koser, Carsten Konrad, Pienie Zwitserlood, Klaus Berger, Marco Hermesdorf, Tilo Kircher, Igor Nenadic, Axel Krug, Bernhard T. Baune, Katharina Dohm, Ronny Redlich, Nils Opel, Volker Arolt, Tim Hahn, Xiaoyi Jiang, Udo Dannlowski, Dominik Grotegerd

**Affiliations:** 1 Department of Psychiatry, University of Münster, Albert Schweitzer-Campus 1, A9, 48149 Münster, Germany; 2 Department of Computer Science, University of Münster, Einsteinstraße 62, 48149 Münster, Germany; 3 Department of Psychiatry and Psychotherapy, Agaplesion Diakonieklinikum, 27356 Rotenburg, Germany; 4 Department of Psychology, University of Münster, Fliednerstraße 21, 48149 Münster, Germany; 5 Department of Epidemiology and Social Medicine, University of Münster, Albert Schweitzer-Campus 1, D3, 48149 Münster, Germany; 6 Department of Psychiatry and Psychotherapy, University of Marburg, Marburg, Germany; 7 Department of Psychiatry, Melbourne Medical School, The University of Melbourne, Parkville, VIC Australia; 8 The Florey Institute of Neuroscience and Mental Health, The University of Melbourne, Parkville, VIC Australia

**Keywords:** Predictive markers, Neuroscience

Correction to: *Neuropsychopharmacology* 10.1038/s41386-020-0666-3, published online 9 April 2020

The article “Biological sex classification with structural MRI data shows increased misclassification in transgender women”, written by Claas Flint, Katharina Förster, Sophie A. Koser, Carsten Konrad, Pienie Zwitserlood, Klaus Berger, Marco Hermesdorf, Tilo Kircher, Igor Nenadic, Axel Krug, Bernhard T. Baune, Katharina Dohm, Ronny Redlich, Nils Opel, Volker Arolt, Tim Hahn, Xiaoyi Jiang, Udo Dannlowski and Dominik Grotegerd, was originally published Online First without Open Access. After publication in volume 45, issue 10, page 1758–1765 the author decided to opt for Open Choice and to make the article an Open Access publication. Therefore, the copyright of the article has been changed to © The Author(s) 2020 and the article is forthwith distributed under the terms of the Creative Commons Attribution 4.0 International License, which permits use, sharing, adaptation, distribution and reproduction in any medium or format, as long as you give appropriate credit to the original author(s) and the source, provide a link to the Creative Commons licence, and indicate if changes were made.

The images or other third party material in this article are included in the article’s Creative Commons licence, unless indicated otherwise in a credit line to the material. If material is not included in the article’s Creative Commons licence and your intended use is not permitted by statutory regulation or exceeds the permitted use, you will need to obtain permission directly from the copyright holder.

To view a copy of this licence, visit http://creativecommons.org/licenses/by/4.0/.

The original article has been corrected.

