# Supplementary material for: Biological sex classification with structural MRI data shows increased misclassification in transgender women
Source: Neuropsychopharmacology. 2020 Apr 9;45(10):1758–65. doi: 10.1038/s41386-020-0666-3 (PMC7419542; doi:10.1038/s41386-020-0666-3)
Supplement: Supplementary file 1 — Supplemental Material [file 41386_2020_666_MOESM1_ESM.docx]

| Table S1. *Descriptive statistics of the trainings and validation samples* | | | |
| --- | --- | --- | --- |
|  | Male | Female | Significance test |
| **Muenster Neuroimaging Cohort** | | | |
| Healthy Controls (*N_male_*=278; *N_female_*=388) | | | |
| Age | 36.4 (11.7) | 35.8 (12.6) | *F*(1,665)=0.4917, *p*=.480 |
| Major Depressive Disorder (*N_male_*=121; *N_female_*=164) | | | |
| Age | 37.4 (11.3) | 38.1 (12.4) | *F*(1,284)=0.253, *p*=.615 |
| BDI | 23.4 (9.5) | 26.5 (10.9) | *F*(1,274)=6.212, *p*=.013 |
| HDRS-17 | 18.5 (3.8) | 19.5 (4.5) | *F*(1,187)=2.739, *p*=.100 |
| IQ (MWBT) | 111.5 (13.3) | 110.9 (14.1) | *F*(1,267)=2.739, *p*=.765 |
| **BiDirect** | | | |
| Healthy Control Group (*N_male_*=217; *N_female_*=217) | | | |
| Age | 51.3 (8.1) | 53.0 (8.0) | *F*(1,433)=4.481, *p*=.035 |
| Major Depressive Disorder (*N_male_*=235; *N_female_*=356) | | | |
| Age | 48.1 (7.4) | 49.6 (7.3) | *F*(1,590)=5.612, *p*=.019 |
| HDRS-17 | 12.6 (6.8) | 14.3 (6.5) | *F*(1,587)=10.160, *p*=.002 |
| **FOR2107** | | |  |
| Healthy Control Group (*N_male_*=246; *N_female_*=407) | | | |
| Age | 32.6 (11.4) | 32.6 (13.0) | *F*(1,652)=0.001, *p*=.976 |
| Major Depressive Disorder (*N_male_*=195; *N_female_*=333) | | | |
| Age | 36.6 (13.8) | 37.8 (13.4) | *F*(1,527)=0.719, *p*=.397 |
| HDRS-17 | 8.9 (6.7) | 8.5 (6.8) | *F*(1,525)=0.414, *p*=.520 |
| *Note.* Table reports means and standard deviations of the individual cohorts used for the training of the support vector machine. Significance test was univariate ANOVA without covariates. | | | |

| Table S2. *Descriptive statistics of the application sample (transgender and cisgender individuals)* | | | | | |
| --- | --- | --- | --- | --- | --- |
|  | CG men  (*N*=15) | CG women  (*N*=19) | TW pre  (*N*=8) | TW post (*N*=18) | significance test |
| Age | 34  (8.6) | 32  (6.3) | 33.9  (14.1) | 33.1  (31.3) | *F*(3,56)=0.190, *p*=.991 |
| Highest Education | 4.9  (0.9) | 4.8  (0.8) | 5.0  (0.0) | 5.1  (0.5) | *F*(3,56)=0.802, *p*=.498 |
| Depressive Symptoms | 51.6  (9.6) | 42.2  (3.8) | 62.9  (13.2) | 60.4  (11.7) | *F(3,52)=13.415, p<0.001* |
| *Note.* Table reports means and standard deviations of the transgender individuals and controls from a similar measurement period used for the test of the support vector machine in TW. Significance test was univariate ANOVA without covariates. 10 of 26 TW subjects reported psychiatric comorbidity. Depressive symptoms were assessed using the Brief Symptom Inventory (BSI [1]). Two TW-pre individuals were taking antidepressant medication at time of scanning.  TW = transgender women (biological sex male, perceived sex female)  pre/post = before/after cross-sex hormone treatment  Age = age in years  Highest Education = measured according to educational attainment in numbers from 1 = special school to 6 = university degree. | | | | | |

Table S3. *Classification results in the application sample*

|  | validation set 1 | validation set 2 | Fisher’s Exact Test |
| --- | --- | --- | --- |
|  | N correct/incorrect | N correct/incorrect | *p*-value |
| CG men | 131/17 | 479/72 | .7386 |
| CG women | 202/1 | 829/24 | .9955 |

*Note.* Comparison of the distribution of classification results between the first and second validation sets, using Fisher's exact test.

CG = cisgender

| Table S4. *Results of the whole-brain analysis* | | | | | | | |
| --- | --- | --- | --- | --- | --- | --- | --- |
| compared groups | region | *TFCE* | *p-FWE* | *k* | *x* | *y* | *z* |
| TW-pre > TW-post | R medial cingulate cortex and caudate nucleus | 1643.35 | .005 | 15117 | 27 | -9 | 16 |
|  | L caudate nucleus | 1514.98 | .005 | 13228 | -22 | 18 | 12 |
|  | L precentral and middle frontal gyrus | 766.08 | .029 | 470 | -36 | 4 | 54 |
|  | L precuneus | 743.23 | .029 | 651 | -14 | -56 | 52 |
|  | L postcentral gyrus | 668.59 | .040 | 410 | -63 | -14 | 39 |
|  | R cerebellum | 632.84 | .045 | 505 | 48 | -54 | -27 |
|  | R cerebellum | 611.18 | .047 | 258 | 40 | -38 | -46 |
| TW-pre > CG-women | L precuneus, R medial cingulate Cortex, L + R lingual gyrus | 982.71 | <.001 | 108742 | 22 | -3 | 15 |
|  | L cerebellum | 328.77 | .003 | 4657 | -30 | -33 | -50 |
|  | R precentral, frontal inferior gyrus | 127.46 | .041 | 360 | 62 | 16 | 27 |
| TW-post > CG-women | R calcarine/lingual gyrus, precuneus | 1312.08 | <.001 | 3875 | 4 | -50 | 3 |
|  | L cuneus, superior occipital gyrus | 605.11 | .023 | 498 | -10 | -96 | 36 |
| TW-post < CG-women | R postcentral gyrus | 1057.71 | .026 | 562 | 45 | -21 | 32 |
| TW-pre > CG-men | L caudate nucleus, putamen, hippocampus | 745.18 | .009 | 3018 | -21 | 18 | 9 |
|  | R caudate nucleus, putamen | 713.97 | .010 | 2336 | 28 | -4 | 16 |
|  | R Precuneus, Mid Cingulum | 673.63 | .013 | 3185 | 8 | -28 | 39 |
|  | L Pre-, Postcentral | 654.18 | .015 | 822 | -69 | -15 | -32 |
|  | R Hippocampus, Parahippocampus | 567.93 | .028 | 713 | 20 | -8 | -30 |
|  | R Calcarine, Lingual gyrus | 528.98 | .002 | 416 | 26 | -75 | 3 |
| TW-post < CG-men | L middle temporal lobe, cerebellum  R middle temporal lobe, cerebellum | 939.61 | <.001 | 111736 | 32 | -56 | -38 |
|  | R precentral and frontal inferior gyrus | 260.12 | .016 | 628 | 57 | 6 | 12 |
| *Note.* For reasons of brevity only significant clusters > k = 300 voxels are reported, we did not calculate a contrast comparing cisgender men and women. The reported significant clusters resulted from group comparisons within a full factorial model corrected for total intracranial volume, age and sexual orientation.  *Abbreviations:* TW = Transgender Women, CG = Cisgender, pre = before hormone treatment, post = after hormone treatment  Coordinates are reported within MNI-space | | | | | | | |

| Table S5. *Comorbidities of transgender individuals.* | | |
| --- | --- | --- |
|  | TW pre  (*N*=2) | TW post  (*N*=8) |
| Substance abuse | 1 | 1 |
| Eating Disorder |  | 1 |
| Obsessive-compulsive Disorder |  | 1 |
| Anxiety Disorder | 1 | 1 |
| Major Depressive Disorder | 2 | 6 |
| *Note.* Psychiatric comorbidities were reported for 10 transgender women.  TW = transgender woman  pre/post = before/after cross-sex hormone treatment | | |

| Table S6. *Classification results separated by scanner features and site* | | | | | | |
| --- | --- | --- | --- | --- | --- | --- |
|  | FOR2107 Muenster | FOR2107 Marburg | MNC Muenster | | BiDirect Muenster | Fisher’s Exact Test  *p*-value |
| first validation sample CG-men | | | | | | .131 |
| correctly classified | 14 | 25 | | 44 | 48 |  |
| incorrectly classified | 2 | 6 | | 7 | 2 |  |
| first validation sample CG-women | | | | | | .586 |
| correctly classified | 26 | 54 | | 84 | 38 |  |
| incorrect classified | 0 | 1 | | 0 | 0 |  |
| second validation sample CG-men with MDD | | | | | | .101 |
| correctly classified | 66 | 107 | | 103 | 203 |  |
| incorrectly classified | 3 | 19 | | 18 | 32 |  |
| second validation sample CG-women with MDD | | | | | | .882 |
| correctly classified | 109 | 213 | | 160 | 347 |  |
| incorrectly classified | 4 | 7 | | 4 | 9 |  |
| *Note.* Table shows frequencies of correctly and incorrectly classified CG-women and –men for all validation samples that used different scanners. The third validation sample was measured with the same MRI-sequence as MNC Muenster.  *Abbreviations:* CG = cisgender, MDD = Major Depressive Disorder | | | | | | |

| Table S7. *ROI analysis in the restricted transgender sample without psychiatric comorbidities* | | | | | | |
| --- | --- | --- | --- | --- | --- | --- |
|  | *k* | *p-FWE* | *TFCE* | *x* | *y* | *z* |
| **Insula** | | | | | | |
| TW-pre>post |  | n.s. |  |  |  |  |
| TW-pre<post |  | n.s. |  |  |  |  |
| TW-pre>CG-women | 642 | .002 | 13.70 | 40 | 20 | -8 |
|  | 107 | .014 | 2.14 | -24 | 21 | -15 |
| TW-pre<CG-women | 471 | .001 | 121.60 | -40 | -3 | 0 |
| TW-pre>CG-men | 2267 | <.001 | 73.19 | -32 | -8 | 12 |
|  | 1271 | <.001 | 19.90 | 38 | -20 | 3 |
|  | 620 | .001 | 3.18 | 46 | 22 | -4 |
| TW-pre<CG-men |  | n.s. |  |  |  |  |
| TW-post>CG-women | 1058 | <.001 | 4.76 | 40 | 21 | 8 |
|  | 479 | .001 | 1.11 | -33 | 26 | 4 |
| TW-post<CG-women | 524 | <.001 | 357.70 | -40 | -8 | 2 |
| TW-post>CG-men | 2427 | <.001 | 24.46 | -32 | 3 | 12 |
|  | 1309 | <.001 | 9.54 | 48 | -10 | 3 |
|  | 130 | <.001 | 5.41 | 45 | 24 | -3 |
|  | 200 | <.001 | 2.11 | 33 | 16 | -21 |
| TW-post<CG-men |  | n.s. |  |  |  |  |
| **Putamen** | | | | | | |
| TW-pre>post | 1179 | .005 | 445.23 | -21 | 16 | 8 |
|  | 519 | .008 | 355.78 | 24 | 6 | 15 |
| TW-pre<post |  | n.s. |  |  |  |  |
| TW-pre>CG-women | 1848 | <.001 | 99.88 | 26 | 6 | 15 |
|  | 2006 | <.001 | 84.36 | -20 | 21 | -3 |
| TW-pre<CG-men |  | n.s. |  |  |  |  |
| TW-pre>CG-men | 2351 | <.001 | 266.62 | -21 | 15 | 8 |
|  | 2130 | <.001 | 155.27 | 28 | -3 | 15 |
| TW-post>CG-women |  | n.s. |  |  |  |  |
| TW-post<CG-women | 324 | .002 | 134.84 | -15 | 9 | 3 |
| TW-post>CG-men | 272 | <.001 | 24.46 | -32 | 3 | 12 |
|  | 215 | <.001 | 2.10 | -28 | 6 | 10 |
|  | 119 | <.001 | 0.52 | 34 | 2 | 4 |
| TW-post<CG-men |  | n.s. |  |  |  |  |
| *Note.* Table shows significant clusters > *k* = 100 voxel for reasons of brevity. Transgender individuals that showed comorbidities according to the Structured Clinical Interview based on DSM-IV-criteria were excluded from the analysis leaving *N* = 6 TW-pre and *N* = 10 TW-post.  *Abbreviations:*  TW-pre = transgender women before cross-sex hormone treatment  TW-post = transgender women after cross-sex hormone treatment  *TFCE* = statistic of the non-parametric approach using threshold-free cluster enhancement  *x, y, z =* coordinates according to MNI-space  *k* = clustersize  n.s. = not significant, k>100 | | | | | | |

| Table S8. *Results of the whole-brain analysis (transgender individuals without psychiatric comorbidities)* | | | | | | | |
| --- | --- | --- | --- | --- | --- | --- | --- |
| compared groups |  | *TFCE* | *p-FWE* | *k* | *x* | *y* | *z* |
| TW-pre > TW-post |  | 1831.52 | .037 | 137 | -21 | -33 | 16 |
| TW-pre > CG-women |  | 2542.38 | <.001 | 124710 | -18 | -34 | 15 |
|  |  | 41.84 | .030 | 603 | -63 | -12 | -38 |
| TW-pre > CG-men |  | 1124.13 | <.001 | 227945 | -26 | -21 | -6 |
| TW-post > CG-men |  | 745.10 | <.001 | 192937 | -18 | -75 | 42 |
|  |  | 21.28 | .003 | 120 | 48 | -30 | -16 |
| *Note.* For reasons of brevity only significant clusters > k = 100 voxels are reported, we did not calculate a contrast comparing cisgender men and women. The reported significant clusters resulted from group comparisons within a full factorial model corrected for total intracranial volume, age and sexual orientation. TW participants with psychiatric comorbidities (N=10) were excluded from the analysis  *Abbreviations:* TW = Transgender Women, CG = Cisgender, pre = before hormone treatment, post = after hormone treatment  Coordinates are reported within MNI-space | | | | | | | |

**Supplementary Methods S1:**

**Images and Structural Preprocessing:**

T1-weighted high-resolution anatomical images of the MNC and TSS were acquired at a 3T MRI (Gyroscan Intera 3T, Philips Medical Systems, the Netherlands) using a three-dimensional fast gradient echo sequence (turbo field echo), repetition time=7.4ms, echo time=3.4ms, flip angle=9°, two signal averages, inversion pre-pulse every 814.5ms, acquired over a field of view of 256 (feet-head)x204 (anterior-posterior) x 160mm³ (right-left), frequency encoding in feet to head direction, phase encoding in anterior-posterior and right-left direction, reconstructed to voxels of 0.5×0.5×0.5mm³ [2,3].

The 3D T1-weighted turbo field echo images of the BD study were collected in the same scanner with repetition time=7.26ms, echo time=3.56, 9° flip angle, 160 sagittal slices, matrix dimension 256 x 256, FOV=256 x 256mm², 2mm slice thickness (reconstructed to 1mm) resulting in a voxel size of 1x1x1mm³.

The FOR2107 study was conducted at two different sites [4]. In Münster, data were collected with a 3T Siemens PRISMA using 3D T1-weighted magnetization prepared rapid acquisition gradient echo (MPRAGE) with repetition time=1900ms, echo time=2.28ms, inversion time=900ms, 8° flip angle, 192 sagittal slices, 0mm slice gap, resulting in a voxel size of 1x1x1mm³. In Marburg, data were collected in a 3T Siemens Magnetom Trio Tim syngo MR B17 using a 3D T1-weighted magnetization prepared rapid acquisition gradient echo (MPRAGE) with repetition time=1900ms, echo time=2.26ms, inversion time=900ms, 9° flip angle, 176 sagittal slices, 0.5mm slice lap, resulting in a voxel size of 1x1x1mm³.

The structural images were preprocessed using the CAT12-toolbox [5] (version r1184) in all four cohorts (MNC, FOR2107, BiDirect, TSS) following published protocols [6]. For the univariate analysis, images were additionally smoothed with a Gaussian kernel of 8mm full width half maximum (FWHM). Absolute threshold masking with a threshold value of 0.1 was used for all univariate second-level analyses (http://www.neuro.uni-jena.de/cat12/CAT12-Manual.pdf). We carefully checked the sample for poor image quality detected by visual inspection and with the check homogeneity using covariance function implemented in CAT12.

**Supplementary Methods S2:**

**Univariate Analysis:**

The TSS sample (TW group and matched CG controls, supplementary table 2) were used in the univariate analysis. Statistical parametric mapping (SPM12, Wellcome Trust Centre for Neuroimaging, London, http://www.fil.ion.ucl.ac.uk/spm/) was used for univariate gray matter analysis. The putamen and insula were defined as a priori regions of interest (ROIs) using the aal-atlas [6] implemented in the Wake Forest University Pickatlas (http://fmri.wfubmc.edu/software/PickAtlas). We investigated the relationship between groups (CG-men, -women, TW-pre, -post-CHT) and gray-matter volume with an ANCOVA, with age, total intracranial volume and sexual orientation as nuisance regressors in all analyses. Sexual orientation was indicated by the participants as a continuous variable (0-100, 0 indicating homosexuality, 50 indicating bisexuality and 100 indicating heterosexuality). Participants could choose each number between 0 and 100. The terminology was chosen according to the natal biological sex of TW, i.e. homosexuality indicated sexual interest in men.

We calculated a priori defined *t*-contrasts according to our hypothesis: CG-men>women, CG-men>TW-pre, TW-pre>CG-women, CG-men>TW-post, TW-post>CG-women and TW-pre>TW-post. An additional whole brain analysis further explored possible regions with volume differences between the groups. Family-wise error correction with p < .05 was used in order to correct for alpha inflation. To determine statistical significance of putative clusters in each of the two bilateral ROIs (insula, putamen) and the whole brain analysis, after applying the non-parametric approach of Threshold-Free Cluster Enhancement as implemented in the TFCE toolbox (http://dbm.neuro.uni-jena.de/tfce, version 167) we conducted 5000 permutations per test.

**Supplementary Results & Discussion S1:**

Basal testosterone was significantly associated with classification as male (r = .437, p = .014) across both TW groups, while progesterone only showed a tendency towards a significant association with classification accuracy (r = .274, p = .09). Looking at TW-pre-CHT and -post-CHT separately, TW-pre CHT showed no significant association (r = .218, p = .302), while TW-post-CHT showed a tendency towards a significant association between basal testosterone concentration and classification as male (r = .392, p =.06).

The classification performance was significantly associated with the amount of basal testosterone measured in TW. However, this association alone is not sufficient to draw any conclusions on the effect of CHT on classification performance, since we did not directly measure the amount of estradiol and anti-androgens that were taken. For instance, it is possible that TW-pre-CHT contain lower levels of basal testosterone without taking any medication which might also increase the feeling of incongruence between natal sex and perceived gender (for a review [7]).

**References Supplemental Materials**

1. Geisheim C, Hahlweg K, Fiegenbaum W, Frank M, Schröder B, Witzleben I von. Das Brief Symptom Inventory (BSI) als Instrument zur Qualitätssicherung in der Psychotherapie. Http://DxDoiOrg/101026//0012-192448128. 2002. 1 January 2002. https://doi.org/10.1026//0012-1924.48.1.28.

2. Dannlowski U, Kugel H, Grotegerd D, Redlich R, Suchy J, Opel N, et al. NCAN cross-disorder risk variant is associated with limbic gray matter deficits in healthy subjects and major depression. Neuropsychopharmacology. 2015;40:2510–2516.

3. Dannlowski U, Kugel H, Grotegerd D, Redlich R, Opel N, Dohm K, et al. Disadvantage of social sensitivity: Interaction of oxytocin receptor genotype and child maltreatment on brain structure. Biol Psychiatry. 2015:1–8.

4. Vogelbacher C, Möbius TWD, Sommer J, Schuster V, Dannlowski U, Kircher T, et al. The Marburg-Münster Affective Disorders Cohort Study (MACS): A quality assurance protocol for MR neuroimaging data. Neuroimage. 2018;172:450–460.

5. Gaser C. Manual Computational Anatomy Toolbox- cat12. Version 184. cat12: http://dbm.neuro.uni-jena.de/cat.

6. Tzourio-Mazoyer N, Landeau B, Papathanassiou D, Crivello F, Etard O, Delcroix N, et al. Automated anatomical labeling of activations in SPM using a macroscopic anatomical parcellation of the MNI MRI single-subject brain. Neuroimage. 2002;15:273–289.

7. Nguyen HB, Loughead J, Lipner E, Hantsoo L, Kornfield SL, Epperson CN. What has sex got to do with it? the role of hormones in the transgender brain. Neuropsychopharmacology. 2018;0:1–16.
