# Supplementary figures and images for: Biological sex classification with structural MRI data shows increased misclassification in transgender women
Source: Neuropsychopharmacology. 2020 Apr 9;45(10):1758–65. doi: 10.1038/s41386-020-0666-3 (PMC7419542; doi:10.1038/s41386-020-0666-3)

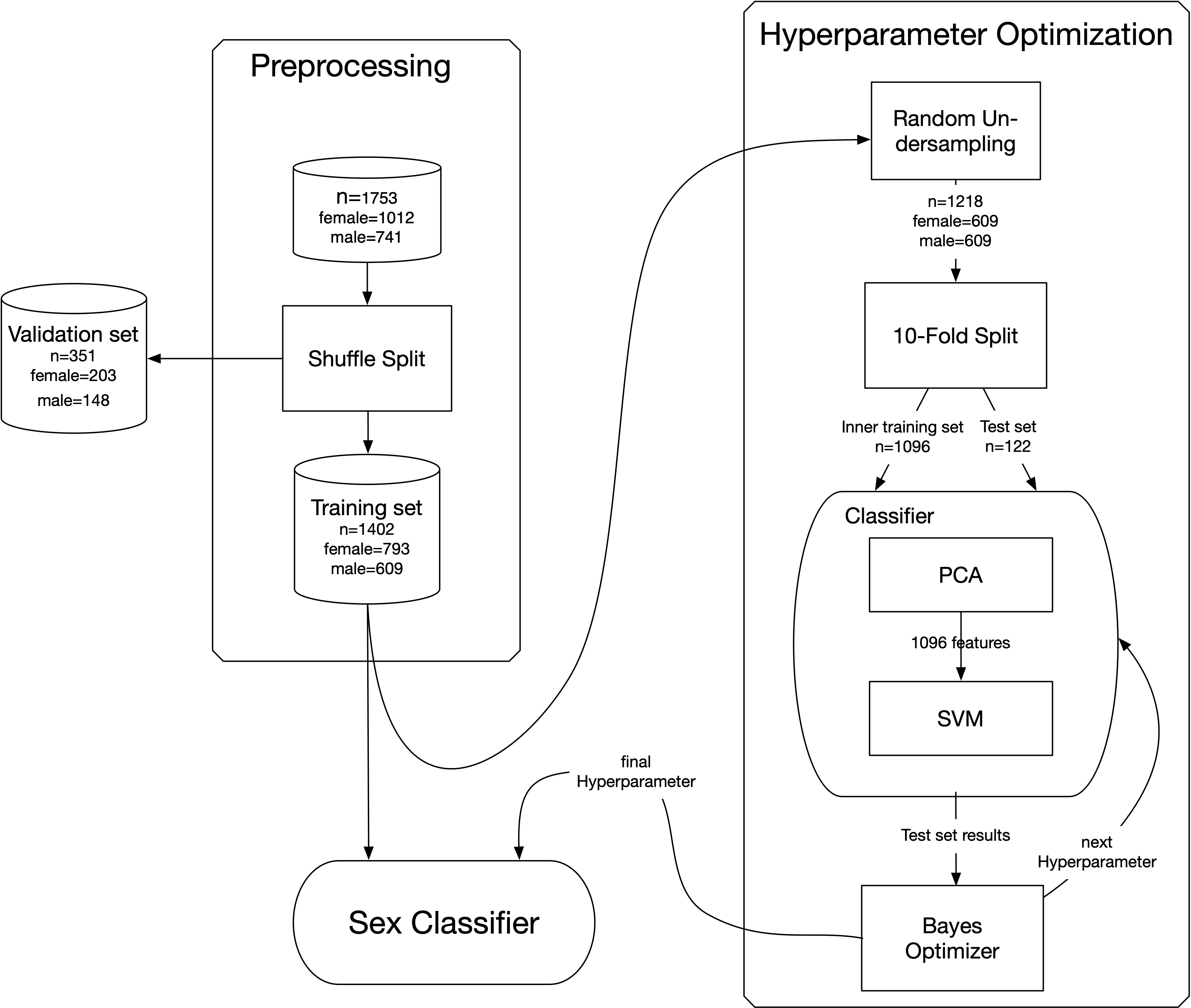

Supplement: Supplementary file 2 — Figure S1 [file 41386_2020_666_MOESM2_ESM.jpg]
